# Addressing the gaps in mental health care for internally displaced persons

**DOI:** 10.7189/jogh.10.010346

**Published:** 2020-06

**Authors:** Sandeep R Sabhlok, Vivian Pender, Elizabeth Mauer, Michael S Lipnick, Gunisha Kaur

**Affiliations:** 1 Department of Anesthesiology, Weill Cornell Medical College, New York, New York, USA; 2 Department of Anesthesia & Perioperative Care, University of California – San Francisco, San Francisco, California, USA; 3 Department of Psychiatry, Weill Cornell Medical College, New York, New York, USA; 4 Department of Psychiatry, Columbia University, New York, New York, USA; 5 Division of Biostatistics & Epidemiology, Weill Cornell Medical College, New York, New York, USA

Exposure to traumatic events such as forced migration contributes to sustained deleterious effects on individual mental health. Between 2010 and 2018, the number of internally displaced persons (IDPs) has nearly tripled from approximately 15 million to 41 million people, making up more than 58% of the worldwide displaced population [1]. These numbers continue to rise with ongoing tensions in South and Southeast Asia, civil unrest in the Middle East, and ethnic conflict in many parts of Africa.

IDPs are forced to flee their habitual residence, but unlike refugees, they have not crossed an internationally recognized border. IDPs are an extremely vulnerable group since they typically remain under the protection of their own government even if their government is the reason behind their displacement. Those fleeing their homes face uncertain circumstances and are often subject to extreme adversity, such as violence, torture, forced detention, separation from or loss of family members, and limited access to adequate health and hygiene resources.

A meta-analysis of over 80 000 displaced civilians found a prevalence rate of approximately 30% for both depression and posttraumatic stress disorder (PTSD) in conflict affected areas across 40 different countries [2]. As a comparison, the prevalence rate for mental disorders in high-income countries is approximately 19% [3]. Mental health services are already significantly limited at baseline in low- and middle-income countries (LMICs), where approximately 74% of IDPs reside [1,4]. Extrapolating from these statistics, up to 9.2 million IDPs in LMICs currently have or are at risk for developing depression or PTSD.

**Figure 1:**
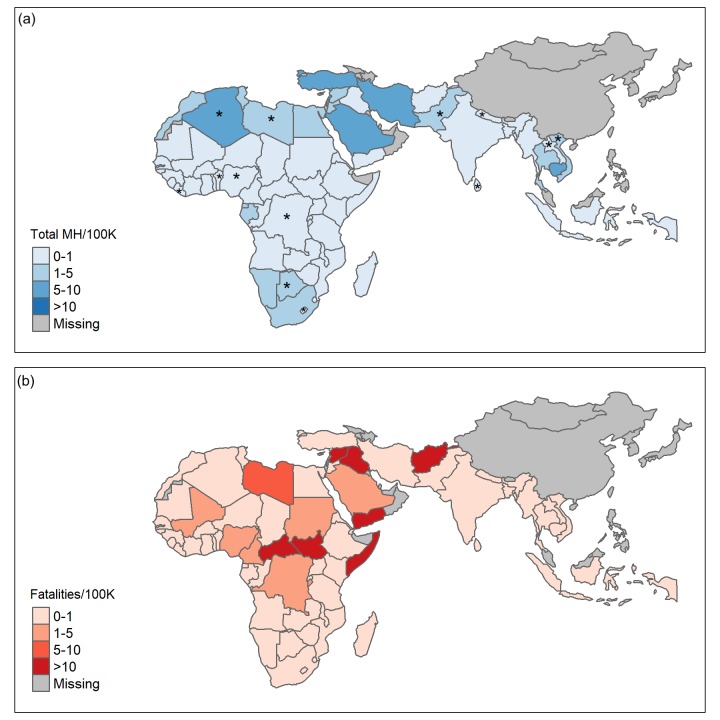
Comparing the mental health workforce with the number of fatalities from ethnic violence in the countries within Africa and Asia. **Panel A.** Total mental health (MH) workforce per 100 000 people. Includes psychiatrists, psychologists, and social workers. Source: 2017 WHO Mental Health Atlas [5]. Asterisks indicate that MH workforce of these countries were not available from the 2017 WHO Mental Health Atlas and data was taken from the 2014 WHO Mental Health Atlas. **Panel B.** Total number of fatalities from ethnic violence in these same countries. Includes violence against unarmed civilians from organized armed groups and remote violence of one-sided violence in which explosions, bombs, or other devices are used to incite conflict. Source: 2017 Armed Conflict Location & Event Data (ACLED) Project [6]. MH workforce of these countries not available from the 2017 WHO Mental Health Atlas and data was taken from the 2014 WHO Mental Health Atlas.

Combining data from geographic information systems (GIS) with the WHO Mental Health Atlas, we mapped the fatalities from ongoing ethnic conflict against the availability of mental health providers (**Figure 1**). This GIS data was obtained using the Armed Conflict Location and Event Database (ACLED), an up-to-date tracker of ethnic and politically driven violence across 68 countries within Africa, Asia, and the Middle East [6]. These maps reveal substantial hot spots where vulnerable populations are left without access to trained mental health providers. The eight countries with the most fatalities from ethnic violence shown in **Figure 1** account for over 42% of the global IDP population. However, there is on average 0.75 mental health providers per 100 000 individuals in these countries compared to a worldwide median of 9 per 100 000 individuals [5].

Without effective screening and treatment, those with undiagnosed mental illness after a period of displacement are vulnerable to suicide, homicide, substance abuse, and human rights abuses [4]. For IDPs within LMICs, these risks are compounded by the combination of persistent violent conflict and a lack of mental health infrastructure [7]. This sets up a vicious cycle between mental illness, social exclusion, unemployment and poverty with effects transcending generations [8].

**Figure:**
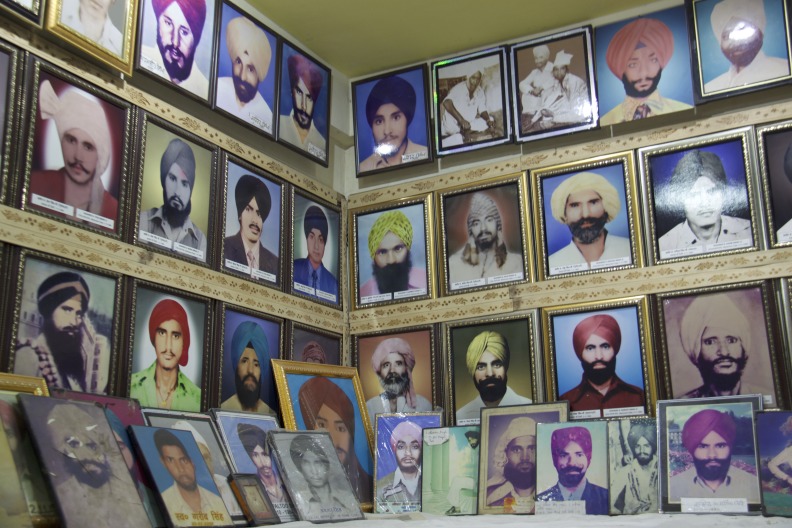
Photo: victims that are within the Gurudwara (Sikh Temple) in the Tilak Vihar Neighborhood of Delhi (from Sandeep Sabhlok’s own collection, used with permission).

As a case study of this, we conducted interviews on a population of women in India displaced as a result of religious and politically motivated violence over three decades ago. On 31 October 1984, Sikh bodyguards assassinated the prime minister of India, Indira Gandhi. Over the proceeding days, a series of organized pogroms occurred across India, most notoriously in Delhi. Now known as the 1984 Anti-Sikh Riots, politically backed mobs massacred an estimated 3000 Sikh men and boys. Fifty thousand Sikhs were displaced, including approximately 1300 new widows within Delhi [9]. Many of these widows were eventually placed into government housing in the Tilak Vihar neighborhood of Delhi, known colloquially as the “Widow Colony.” Despite ethnographic accounts highlighting high rates of substance abuse and suicide in this community [9], no studies to date have examined any of the health consequences of the trauma experienced by these women. Our investigation was the first ever study and documentation of mental illness in internally displaced persons within the Widow Colony.

Using two validated screening tools, the Patient Health Questionnaire (PHQ-9) [10] and the PTSD Checklist for DSM-5 (PCL-5) [11], we interviewed 25 widows. 57% of those screened were found positive for at least moderate depression and 67% were found positive for PTSD. These women described flashbacks, uncontrollable anger, and a sense of disconnect from their family and community. Many were unaware their symptoms may warrant a medical diagnosis, assuming these emotions were due to *karma*, or their destiny due to actions in a past life. Even though many women expressed gratitude in being able to share the details surrounding their trauma, few reported any psychological interventions by local physicians or health care workers. The sole woman diagnosed and under treatment for depression reported, “The medications saved me and my family.” When physicians in the community were interviewed, they harbored disbelief regarding the women’s ongoing psychological struggles, which subsequently imbued significant mistrust of physicians amongst the women of the Widow Colony.

To ameliorate the mental health consequences of persistent unaddressed trauma, as exemplified by our study, a multi-faceted approach is required. Experts suggest that this entails building clinical capacity, creating novel treatment models, working within communities to overcome barriers to equitable care, and collaborating to advance the global mental health research agenda [12]. With ongoing global conflict, incorporating trauma informed mental health care will also become necessary, especially in LMICs where a significant shortage of mental health resources exists. By creating strong partnerships and policies in areas of conflict or human rights abuses, we can reinforce the mental health needs of the forcibly displaced population as a public health priority.
